# Copy Number and Prevalence of Porcine Endogenous Retroviruses (PERVs) in German Wild Boars

**DOI:** 10.3390/v12040419

**Published:** 2020-04-08

**Authors:** Luise Krüger, Milena Stillfried, Carolin Prinz, Vanessa Schröder, Lena Katharina Neubert, Joachim Denner

**Affiliations:** 1 Robert Koch Fellow, Robert Koch Institute, 13353 Berlin, Germany; KruegerL@rki.de (L.K.); carolin_runge@arcor.de (C.P.); vanessaj.schroeder@googlemail.com (V.S.); ln337@cam.ac.uk (L.K.N.); 2 Department of Ecological Dynamics, Leibniz Institute for Zoo and Wildlife Research, 10315 Berlin, Germany; milena.stillfried@web.de

**Keywords:** porcine endogenous retroviruses (PERV), wild boars, copy number, PERV-C

## Abstract

Porcine endogenous retroviruses (PERVs) are integrated in the genome of pigs and are transmitted like cellular genes from parents to the offspring. Whereas PERV-A and PERV-B are present in all pigs, PERV-C was found to be in many, but not all pigs. When PERV-C is present, recombination with PERV-A may happen and the PERV-A/C recombinants are characterized by a high replication rate. Until now, nothing has been known about the copy number of PERVs in wild boars and little is known about the prevalence of the phylogenetically youngest PERV-C in ancient wild boars. Here we investigated for the first time the copy number of PERVs in different populations of wild boars in and around Berlin using droplet digital PCR. Copy numbers between 3 and 69 per genome have been measured. A lower number but a higher variability was found compared to domestic pigs, including minipigs reported earlier (Fiebig et al., Xenotransplantation, 2018). The wild boar populations differed genetically and had been isolated during the existence of the Berlin wall. Despite this, the variations in copy number were larger in a single population compared to the differences between the populations. PERV-C was found in all 92 analyzed animals. Differences in the copy number of PERV in different organs of a single wild boar indicate that PERVs are also active in wild boars, replicating and infecting new cells as has been shown in domestic pigs.

## 1. Introduction

Endogenous retroviruses are the result of infection and integration of ancient retroviruses into the germ line of a host. They are widely distributed in many species including humans and play an important role in placentogenesis [1]. In pigs, three types of porcine endogenous retroviruses (PERVs) have been described: PERV-A and PERV-B, which are found in all pigs, and PERV-C, which are found in many, but not all pigs. All three viruses belong to the genus gammaretroviruses and they differ in their envelope protein, mainly in the receptor-binding domain and in the receptor usage. They are highly related in the gag and pol sequences but differ in the sequence of the long terminal repeats (LTR), as well as in the age of integration and the prevalence among pigs. PERV-A and PERV-B are able to infect cells from humans and many other species, while PERV-C is an eco-tropic virus infecting only pig cells [2]. PERV-A and PERV-C have the same origin and are younger compared to PERV-B. PERV is approximately 7.6 million years old [3]. PERVs without sequence repeats in the U3 region of the LTR evolved approximately 3.4 million years ago, being the phylogenetically younger structure. The age determined for PERV correlates with the time of separation between pigs (Suidae, Sus scrofa) and their closest relatives, American born peccaries, 7.4 million years ago [3]. PERV-C originated more recently (1.5 to 3.5 million years ago) [4]. While all Suidae members including the warthog and red river hog contained PERV-B, the warthog lacked detectable PERV-A and PERV-C and the red river hog lacked PERV-C [5].

As the result of de novo infection and/or transposition, multiple integrated proviruses are found in the pig genome. The estimate of the exact number of pro-viral copies is difficult, and different methods give different results (for review see [6]). For example, using real-time PCR, between one and 95 copies have been reported. Differences between different pig breeds have also been observed. Using droplet digital PCR (ddPCR), between 46 to 70 copies have been found in four different lines of pig kidney PK-15 cells [7,8] and 26 copies in porcine fetal fibroblast cells [9], as well as 69 copies in Aachen minipigs, 64 copies in Göttingen minipigs, and 59 copies in German landrace pigs [7]. Furthermore, different copy numbers were found in different organs of a single pig, indicating that PERVs are de novo infecting and integrating into pig cell genomes in the living animal [6,7]. 

**Figure 1:**
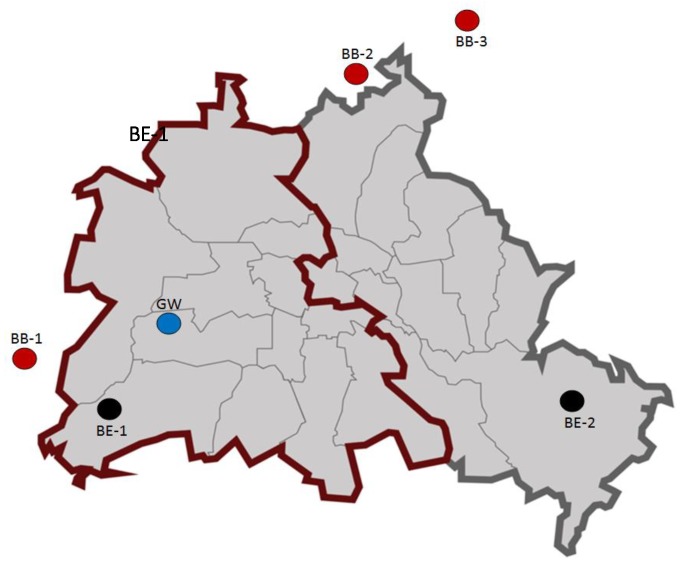
Origin and genetic characterization (Bayesian Analysis of Population Structure, BAPS cluster) of the wild boars tested. The locations are: Berlin Grunewald (GW, blue) (BAPS cluster 1), Berlin Brandenburg (BB-1, -2, -3; red), Berlin East (BE-1, -2, black) (all BAPS cluster 2). The red line indicates the Berlin wall around Berlin West, the gray line indicates the border of East Berlin. Outside Berlin is the state Brandenburg.

As mentioned above, PERV-C was not found in all pigs. The prevalence of PERV-C in various pig breeds in different countries ranged from 6% to 100% (Table 1) [10,11,12,13,14,15,16,17,18,19,20]. Only one previous study by our laboratory found a prevalence of 100% in wild boars near Berlin, Germany [11]; in addition, a single wild boar in the Netherlands was analyzed and no PERV-C was found [20]. To study the prevalence of the phylogenetically youngest PERV-C in ancient wild boars, a greater number of wild boars living in two different urban forests in Berlin as well as in forests in Brandenburg, outside Berlin, were screened. These forests were divided by the Berlin wall which existed in the years 1961 until 1989. This means the wild boars in East and West Berlin had no contact in these years. The animals had been genetically analyzed and based on the analysis of 13 microsatellite loci, samples were grouped into two genetic clusters: animals living in the Grunewald forest, in the former Western part of Berlin, belonged to the Bayesian Analysis of Population Structure (BAPS) cluster 1, while animals in the other regions and mainly in Brandenburg belonged to the BAPS cluster 2 [21] (Figure 1). Therefore, it would be of interest whether the division of the populations for nearly 30 years had an influence on the general copy number in the genome of the pigs and the prevalence of PERV-C. PERV-C is an important topic in xenotransplantation since the presence of the ecotropic PERV-C allows recombination with PERV-A, resulting in human-tropic and highly replication-competent PERV-A/C [22,23,24].

## 2. Materials and Methods 

### 2.1. Animals

Wild boars (*n* = 89) were hunted in the city of Berlin as well as in adjoining parts of the federal State of Brandenburg, Germany, for food production by hunters of the state- and town-owned forests and by special hunters in Berlin. Between 1949 and 1989, two German states, the Federal Republic of Germany (FRG) and the German Democratic Republic (GDR), existed and Berlin was divided by the 167 km long Berlin wall which surrounded the Western part of the city. In total, four genetic clusters of wild boars have been identified based on the analysis of 13 microsatellite loci, two within urban forests that belonged to former West-Berlin (Grunewald and Tegel) and two in the former Eastern part (Köpenick and Brandenburg including Pankow) (Figure 1) [21]. We included samples of one Eastern and one Western cluster in this study: the Grunewald Bayesian Analysis of Population Structure (BAPS) cluster 1 and the Brandenburg BAPS cluster 2 (including also wild boars from Pankow) [21]. All animals were older than one year. Spleens were sampled directly by the scientists in agreement with the hunters between 2011 and 2015, stored frozen at −20 °C and DNA was isolated at the Leibniz Institute. 

In addition, three wild boars were hunted for food production near Berlin in the state Brandenburg in 2019. The heart, liver, spleen, kidney and lung tissues were removed immediately, stored in a refrigerator and DNA was isolated the next day at the Robert Koch Institute. 

The estimated wild boar population size in the sampling area was calculated to be between 5000 and 10,000 animals. Of the population, 2644 animals were shot in Berlin and 89,819 animals were shot in the entire state of Brandenburg in the years 2017/2018 [25].

Three PERV-C positive domestic pigs (German landrace) were used for comparison.

### 2.2. DNA Isolation

DNA from the 89 wild boars hunted between 2011 and 2015 was extracted using the First-DNA all-tissue Kit (Gen-Ial GmbH, Troisdorf, Germany), following the manufacturer’s instructions. DNA was quantified using a Qubit 3.0 Fluorometer (ThermoFischer) and the 260nm/280nm ratio was determined using a NanoDrop ND-1000 (Thermo Fisher Scientific Inc., Worcester, MA, USA) and used for genetic studies including analysis of 13 microsatellite loci [21] and our studies. 

The DNA from tissues of the three animals hunted in the state Brandenburg in 2019 was isolated using the DNeasy blood and tissue kit (Qiagen) according to the manufacturer’s instructions.

### 2.3. Classical PCR

Using a primer pair in the pol region of PERV (PERVpol), all PERV types (PERV-A, PERV-B and PERV-C) were detected [8]; PERV-C was detected using specific primers for the envelope region of PERV-C (Supplemental Table S1) [15]. 

### 2.4. Droplet Digital PCR

Droplet Digital PCR (ddPCR) was performed according to the manufacturer’s instructions (Bio-Rad, Hercules, CA, USA, [http://www.bio-rad.com/de-de/applications-technologies/droplet-digital-pcr-ddpcr-technology?ID=MDV31M4VY]) using a QX200 droplet generator and a QX100 droplet reader (Bio-Rad). Purified genomic DNA (100 ng genomic DNA) was digested with MseI (New England Biolabs, USA) (20U) at 37 °C for 1 h and the restriction enzyme was heat inactivated. The DNA digest was diluted to 5–10 ng/µL for the ddPCR reaction. The ddPCR mix consisted of 10 μL 2× ddPCR Master mix, 1.8 μL of each 10 µM target primers (Supplemental Table S1), 0.5 µl of each 10 µM probes (FAM/HEX), 2.5–10 ng digested DNA and water to a total volume of 20 μL. The following cycling conditions were used: 10min initial enzyme activation at 95 °C, 30 sec denaturation at 94 °C, 1 min annealing and extension at 59 °C (45 cycles) and final 10 min enzyme deactivation at 98 °C using a Master cycler ProS (Eppendorf). The temperature ramp rate was 2 °C per second. To run a gradient, temperatures between 57 and 62 °C were used. To determine the copy number the program Quanta Soft 1.7.4 was used. In order to measure the exact copy number porcine GAPDH or porcine beta actin was used as reference (for primers and probes see Supplemental Table S1).

### 2.5. DNA Agarose Gel Electrophoresis-PCR

This method is like a Southern blot analysis and was used to demonstrate integration of retroviral proviruses into high molecular cellular DNA. Purified cellular wild boar DNA was digested with the restriction enzyme EcoRI (fast digestion, Thermo Fisher), and an agarose gel (0.8%) electrophoresis (70 V, 2 h) was performed. Afterwards the gel was cut in equal fractions, the DNA was isolated from each fraction and tested in a classical PCR using primers specific for PERVpol (Supplemental Figure S1). Whereas in the Southern blot analysis a radioactive probe is used to detect viral sequences in high-molecular DNA fragments with had been obtained by treatment with a restriction enzyme and separated by electrophoresis, here a PCR with two virus-specific primers was used. 

### 2.6. Ethical Statement

All tissue samples were collected from wild boars hunted for food production by hunters of the state- and town-owned forests and by special hunters in Berlin and independently from the project; therefore, no wild boars were harmed or killed for the project.

## 3. Results

### 3.1. PERV Copy Number in Berlin/Brandenburg Wild Boars

PERV-A and PERV-B were found by PCR using specific primers for the PERV-A*env* and PERV-B*env* sequences in all wild boars, therefore their prevalence was 100%. The integration of the proviruses was demonstrated by a new method which is equivalent to the Southern blot analysis; PERV integration was demonstrated by PCR detection of viral sequences in high molecular weight fractions of pig DNA after or without EcoRI digestion and agarose gel electrophoresis (Supplemental Figure S1). 

**Figure 2:**
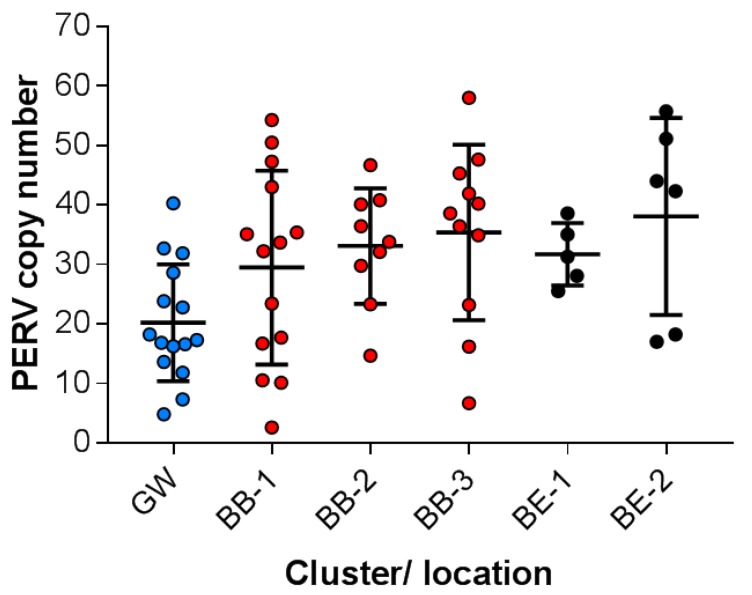
Number of integrated PERV proviruses in genetically different wild boars living in different locations in Berlin and Brandenburg. The animals are sorted by the location and the BAPS cluster. Each point indicates one animal, the median and the standard deviations are shown. Grunewald (GW, blue) (BAPS cluster 1), Berlin Brandenburg (BB-1, -2, -3; red), Berlin East (BE-1, -2, black) (all BAPS cluster 2), see Figure 1.

Using a droplet digital PCR (ddPCR) with a probe corresponding to a pol sequence (Supplemental Table S1) highly conserved among all PERVs and using as a reference the porcine GAPDH, the total copy number of all PERV sequences (including PERV-A, PERV-B and PERV-C) was measured. The ddPCR had been validated before, always showing identical results (Supplemental Figure S2). In the 15 investigated wild boars from the Grunewald (GW) (BAPS cluster 1) sample location, PERV copy numbers between 5 and 40 were found with a median of 20 copies and a standard deviation of 9.5 (Figure 2). All other animals belonged to BAPS cluster 2: in the 14 investigated wild boars of the Berlin-Brandenburg sample location 1 (BB-1), between three and 54 copies were found with a median of 29 copies and a standard deviation of 16. In the nine investigated wild boars of the Berlin-Brandenburg sample location 2 (BB-2), between 15 and 47 copies were found with a median of 35 copies and a standard deviation of 14. In the 11 investigated pigs of the Berlin-Brandenburg sample location 3 (BB-3), between seven and 58 copies were found with a median of 35 and a standard deviation of 14. In the five pigs of the Berlin East sample location 1 (BE-1), between 26 and 39 copies were found with a median of 32 and a standard deviation of five. Finally, in the six animals of the Berlin East sample location 2 (BE-2), between 17 and 56 copies were found with a median of 38 and a standard deviation of 15. This animal group was interesting because two animals had a low copy number (17 and 18) and the others had a high copy number (42–56). In summary, in all animals the copy number varied between three and 56 and the differences in each cluster group were larger compared with the differences between the sample locations (between 29 and 38 copies) (Figure 2). In most cases the differences in the copy number between animals were statistically not significant. However, the differences between wild boars in GW (Grunewald) and BB-3 (Berlin-Brandenburg - 3) (*p* = 0.005) and between GW and BE-1 (Berlin East 1) were significant (*p* = 0.02) (Figure 2). 

### 3.2. PERV Copy Numbers in Different Organs of a Single Pig

**Figure 3:**
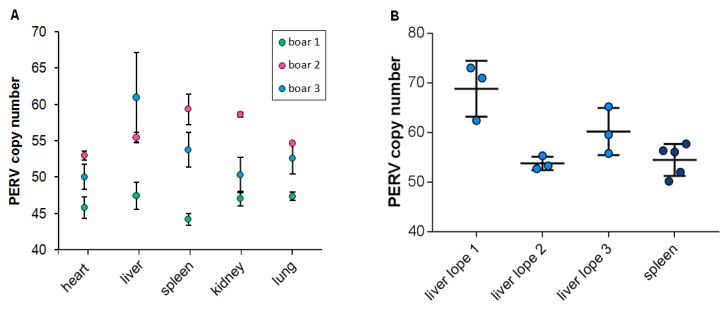
Number of integrated PERV proviruses (**A**) in different organs from three recently hunted wild boars in Brandenburg and (**B**) in three different regions of the liver and in five regions of the spleen of wild boar 3.

It has been shown that in domestic pigs the PERV copy number is different in different organs of a single animal, indicating that PERVs are active and infect new cells and integrate de novo in somatic cell genomes in living pigs [6,7]. In order to analyze whether the same situation can be observed in wild boars, organs from three different wild boars hunted in the state Brandenburg near Berlin were analyzed. Using ddPCR, the heart, liver, spleen, kidney and lung of three animals were analyzed (Figure 3A). In the case of wild boar 1, the copy number varied between 44 and 47 with a median of 46 and a standard deviation of 1.2 for all tested organs. In the case of wild boar 2, the copy number varied between 53 and 59 with a median of 56 and a standard deviation of 2.4. In the case of wild boar 3, the copy number varied between 50 and 61 with a median of 53.5 and a standard deviation of four. A large difference was observed in the case of the liver, with 46 copies in animal 1 and 61 copies in animal 3. The animal with the largest difference in organs was animal 3, ranging from 49 copies in the heart and 61 copies in the liver. Although the number of analyzed animals was small, a great variability was observed in the liver and spleen of all animals and a low variability was found in the heart, reflecting higher cell proliferation and increased de novo integration of PERV in liver and spleen compared to the heart. 

To analyze the difference inside a single organ of a wild boar, the copy number in three different lobes of the liver from three animals and the copy number in five different regions of the spleen of a single animal were studied (Figure 3B). The copy numbers ranged from 44 in one spleen to 62 in one liver (Figure 3A) and from 53 to 74 in different liver lobes (Figure 3B). The difference in the copy number between the liver lobe 1 and liver lobe 2 was significant (*p* = 0.007) (Figure 3B).

### 3.3. Prevalence of PERV-C

**Figure 4:**
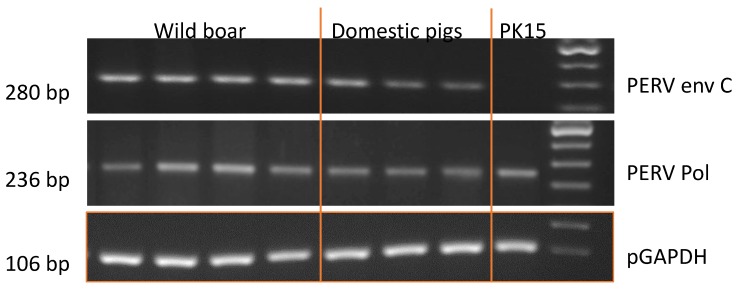
Results of the PCR specific for PERV-C and pol region of PERV (PERVpol) of DNA from wild boars and domestic pigs (German landrace). PK15 cells were used as control and the length of the amplicons is indicated.

Using a classic PCR, PERV-C was detected in all tested 92 wild boars from all sample locations (Figure 4). The primer binding and the identical size of the amplicons indicate that the sequences in domestic pigs (German landrace) and wild boars analyzed here are identical (Figure 4).

## 4. Discussion

This study was performed to answer the question of whether wild boars, which are more ancient compared with domestic pigs, contain less PERV sequences in their genome and whether they harbor PERV-C, the phylogenetically youngest PERV. The main results of the study are: (i) For the first time the number of PERV copies in wild boars was estimated using ddPCR, (ii) the PERV copy number is slightly lower compared with domestic pigs, but differed significantly from animal to animal, (iii) as expected, PERV is integrated in the genome of wild boars, (iv) the PERV copy number in different tissues of a single animal is different, indicating an active replication and de novo integration in the living pigs, and (v) PERV-C was found to be present in all pigs in and around Berlin. 

(i) The copy number of PERVs in different pig breeds but not in wild boars had been analyzed in the past and differences between different pig breeds had been observed (Table 1). In these studies, different methods had been used, mainly real-time PCR, but also Southern blot, PCR titration, fluorescence in situ hybridization (FISH), genome wide sequencing and ddPCR (for review see [6]). Therefore, the reason for the different results may be in the method used and/or the pig breed analyzed. From all methods, ddPCR seems to be the most reliable. One major advantage is that reference standard curves are not required for ddPCR, and bias effects arising from amplification efficiency and PCR inhibitors were reduced. As widely demonstrated in the literature, the ddPCR technique has some other favorable features compared to real-time PCR: it provides absolute quantification based on the principles of sample partitioning and Poisson statistics, thus overcoming the normalization and calibrator issues; it has shown increased precision and sensitivity; it is relatively insensitive to PCR inhibitors and directly provides the result of the analysis expressed as number of copies of target per microliter of reaction [26]. These data, including our own experience with the real-time PCR, demonstrate the superiority of ddPCR when compared to other methods. 

(ii) The PERV copy number in wild boars is lower compared to that in domestic pigs and minipigs but differed significantly from wild boar to wild boar as shown above (Figure 2). Previously, we analyzed the PERV copy number in Göttingen minipigs, which had been used in a preclinical trial transplanting islet cells into cynomolgus monkeys without transmission of PERV [27], and in Aachen minipigs using ddPCR. Forty-five to 93 (mean 64) and 34 to 97 (mean 69) copies, respectively, were found [7]. The copy number in these minipgs is higher compared with the wild boars; the difference between all wild boars studied here (*n* = 59, Figure 2) and Göttingen minipigs studied previously (*n* = 15, Reference [7]) is significant (*p* = 3.78988 × 10^−15^). In a similar situation, studies have shown that in inbred mice the number of murine endogenous proviruses is also higher compared with wild mice [28]. PERV-Cs were found in both Göttingen and Aachen minipigs. Whereas in Göttingen minipigs from Ellegaard no recombinant PERV-A/Cs were found [13], in some Aachen minipigs PERV-A/C was found in the liver and spleen, but not in the peripheral blood mononuclear cells (PBMCs) [29]. In contrast, PERV-A/Cs were found in PBMCs from some Göttingen minipigs bred at the Göttingen university with a high expression of PERVpol and PERV-C [30].

(iii) Although the integration of PERVs into the genome of different pig cell lines such as PK15, ST-IOWA and others, and in PERV-infected human 293 cells has been shown using Southern blot analysis [31], no such experiments had been performed with DNA from wild boars. A PCR analysis of fractions of wild boar DNA after EcoRI digestion and agarose gel electrophoresis showed positive results in high molecular and undigested DNA (Supplemental Figure S1), clearly demonstrating PERV integration in the wild boar genome.

(iv) The fact that we detected different copy numbers of PERV in different tissues of a single animal and even in the same organ indicates an active replication and de novo integration in the living pigs. An endogenous retrovirus behaves usually like a cellular gene in that it is present in all cells of the organism with the same copy number [1]. However, PERV is still active and therefore the copy number is higher in some organs where it replicates well compared to the germline.

Among all species, chicken, mice, and cats are best analyzed concerning the endogenous retroviruses in their genomes. For example, during cat evolution various exogenous retroviruses infected different cat lineages and generated numerous endogenous retroviruses in the host genome, some of which remain replication competent [32]. These viruses also can recombine and integrate de novo. In all these species, eco-tropic viruses were found to be able to infect only cells of their own species, as well as xenotropic or polytropic viruses being able to infect cells of other species. Whereas in these species retroviruses are known to induce tumors, in pigs until now such an association was not reported [1,33]. In addition, in many mammalian species the role of endogenous retroviruses in placentogenesis is well studied, whereas in pigs an involvement of PERVs is still unknown [34].

(v) The fact that all wild boars in and around Berlin are carrying PERV-C is interesting because some domestic pigs of different strains are PERV-C negative (Table 1). This finding confirms our previous report [11] showing that wild boars near Berlin were all PERV-C positive. Currently there is still no information about wild boars in other countries. This also indicates that PERV-C was introduced into these animals a long time ago. The animals in Berlin and Brandenburg represent recent European wild boar S. s. scrofa, a medium-sized, dark to rusty brown-haired subspecies with long and relatively narrow lacrimal bones. The evolutionary history of Sus is best explained by many episodes of interspecific admixture [35]. Pigs were definitively domesticated in an independent long-term process in at least two locations: East Asia and Near East. However, there is a lack of consistency between the genetic and archeological records, and domestic pigs appeared suddenly alongside wild boars in Europe and other places. Once there, hybridization with the local wild boar happened. The genetic differentiation within the modern wild boar, Sus scrofa, is mirrored by significant morphological variation, and 15 subtypes and 80 taxa have been described within S. scrofa [35]. Importantly, wild boars harbor a significant amount of genetic variation not found in domestic populations. This goes in line with the genetic differences observed in the pigs in East and West Berlin [21]. Despite this there is no significant difference in the PERV copy number between the different populations in and around Berlin (Figure 2).

At present it remains unclear whether wild boars can release human-tropic PERVs. The common method to detect this, e.g., co-incubation of mitogen-stimulated pig PBMCs highly susceptible to human 293 cells, could not be used since wild boar PBMCs were not available. However, based on previous studies, it is unlikely that wild boars release infectious particles in this assay because until now positive results were reported only in minipigs [36,37], including one case of Göttingen minipigs, whereas PBMCs from 50 other pigs did not release the virus [31]. In all cases PERV-A/C were released from PBMCs and detected by this assay [31,37,38]

Finally, it should be mentioned that numerous other viruses have been found in wild boars, including suid alphaherpesvirus 1 (pseudorabies virus), a pestivirus causing classical swine fever, porcine reproductive and respiratory syndrome virus (PRRSV), porcine circovirus 2 (PCV2) and many others [38,39,40,41,42,43,44]. It seems likely that the origin of PCV2 infection in wild boars could be through contact with domestic pigs, not least because of the high PCV2 infection rate in pig herds. An only recently detected new circovirus, porcine circovirus 3 (PCV3), was also found in wild boars [42,43]. Most interestingly, PCV2 and PCV3 were also found in up to 50% of both clusters of the wild boars in East and West Berlin analyzed here [44]. 

## 5. Conclusions

This is the first determination of the PERV copy number in wild boars. The number is slightly lower compared with that of domestic pigs. In genetically different populations of wild boars in and around Berlin, which were divided during the existence of the Berlin wall, the copy number of PERVs was nearly the same. Most importantly, the copy number differed from animal to animal. Furthermore, differences in the copy number of PERV in different tissues of a single animal indicate an active replication and de novo integration of PERV in the living pigs. Although domestic pigs originate from ancestors of the wild boars, all wild boars studied here were PERV-C positive whereas some domestic pigs are PERV-C negative. 

## Figures and Tables

**Table 1:** Prevalence of Porcine endogenous retroviruse (PERV)-C in different pig breeds. Wild boars are shown in position 1 and 13.

Number	Pig Breed	Positive Pigs/Tested Pigs	Percentage (%)	Reference
1	Transgenic and non-transgenic, Germany	176/181	97	Dieckhoff et al., 2009 [11]
	Wild boar, Berlin	18/18	100	
2	Farm animals, USA	1/16, 2/16, 14/34, 8/32	6.3–41.2	Pal et al., 2011 [12]
3	Göttingen minipigs	15/15	100	Semaan et al., 2013 [13]
4	Chinese miniature pigs	6/20	30	Liu et al., 2011 [14]
5	Farm animals, German landrace, Germany	14/16	87.5	Kaulitz et al., 2011 [15]
	Genetically modified German landrace	14/15	93.3	
	German landrace ×Duroc×minipig	7/7	100	
9 *	Berkshire	129/191	68	Fujimura et al., 2008 [16]
	Landrace	8/16	50	
	Duroc	26/50	52	
	Large White	9/43	21	
	Miniature, pig	5/6	83	
	Genetically modified triple cross-breed pig	36/36	100	
10	Chinese miniature pigs	113/348	30	Wu et al., 2007 [17]
11	Munich miniature swine (MMS) Troll	4/4	100	Dieckhoff et al., 2007 [18]
12	Miniature swine	17/17	100	Hector et al., 2007 [19]
13 **	Pietran	all tested ***	100	Mang et al. 2001 [20]
	Hampshire	all tested	100	
	Meishan	all tested	100	
	Wild boar	0/1	0	
	Large White	all tested	0	
	Dutch Landrace	all tested	100	

*All animals from Japan, ** all animals from the Netherlands, *** the number of tested animals was not indicated.

## Supplementary Materials

Supplementary materials can be found at https://www.mdpi.com/1999-4915/12/4/419/s1. Figure S1: A, Agarose gel (0.8%) electrophoresis (70 V, 2 hours) of purified DNA from a wild boar, either untreated (lane 1) or treated with EcoRI (fast digest, Thermo Fisher) for 90 min (lane 2, 3). The marker (M) is the GeneRuler 1 kb DNA ladder (Thermo Fisher). After electrophoresis the gel was cut in fractions (1 to 14), from each fraction the DNA was isolated and tested in a PCR using the PERVpol primers. PERV positive (+, ++, +++, ++++) and negative fractions (--) are indicated. In parallel the high-molecular DNA from the untreated DNA (ellipse in lane 1, A) was isolated and analysed in the PCR using PERVpol primers. The result was ++++. B, Results of the PCR testing for PERV using the DNA eluted from gel fractions 1 to 14 and untreated sample (U) as shown in figure A, M, Marker; PK15, positive control, DNA from PK15 cells, the length of the amplicon (236 bp) is marked with an arrow; Table S1: Primers and probes used for the detection of PERV.
