# Supplementary material for: Copy Number and Prevalence of Porcine Endogenous Retroviruses (PERVs) in German Wild Boars
Source: Viruses. 2020 Apr 8;12(4):419. doi: 10.3390/v12040419 (PMC7232352; doi:10.3390/v12040419)
Supplement: Supplementary file 1 [file viruses-12-00419-s001.pdf]

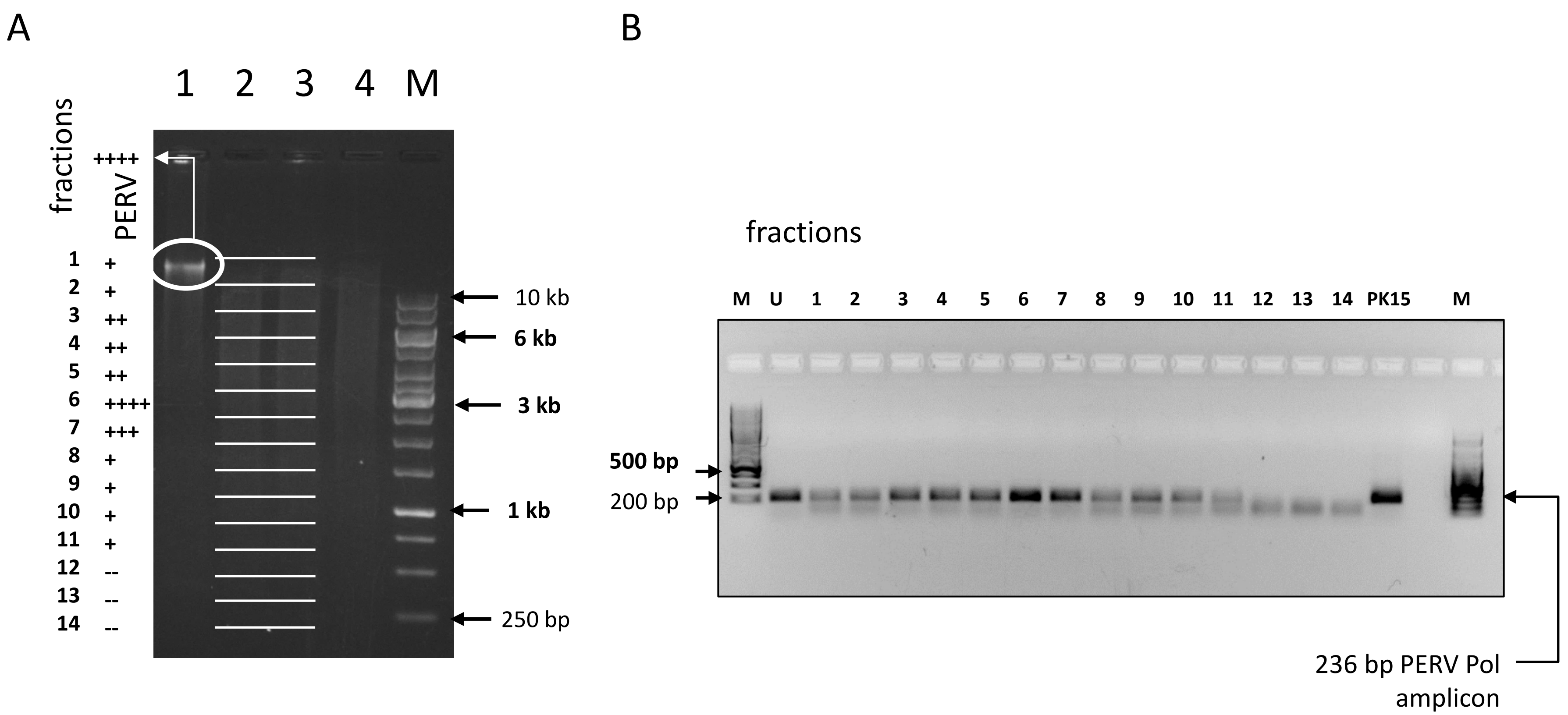

Supplemental Figure 1, A, Agarose gel (0.8%) electrophoresis (70 V, 2 hours) of purified DNA from a wild boar, either untreated (lane 1) or treated with EcoRI (fast digest, Thermo Fisher) for 90 min (lane 2, 3). The marker (M) is the GeneRuler 1 kb DNA ladder (Thermo Fisher). After electrophoresis the gel was cut in fractions (1 to 14), from each fraction the DNA was isolated and tested in a PCR using the PERVpol primers. PERV positive (+, ++, +++, ++++) and negative fractions (--) are indicated. In parallel the high-molecular DNA from the untreated DNA (ellipse in lane 1, A) was isolated and analysed in the PCR using PERVpol primers. The result was ++++.. **B**, Results of the PCR testing for PERV using the DNA eluted from gel fractions 1 to 14 and untreated sample (U) as shown in figure A, M, Marker; PK15, positive control, DNA from PK15 cells, the length of the amplicon (236 bp) is marked with an arrow.

A

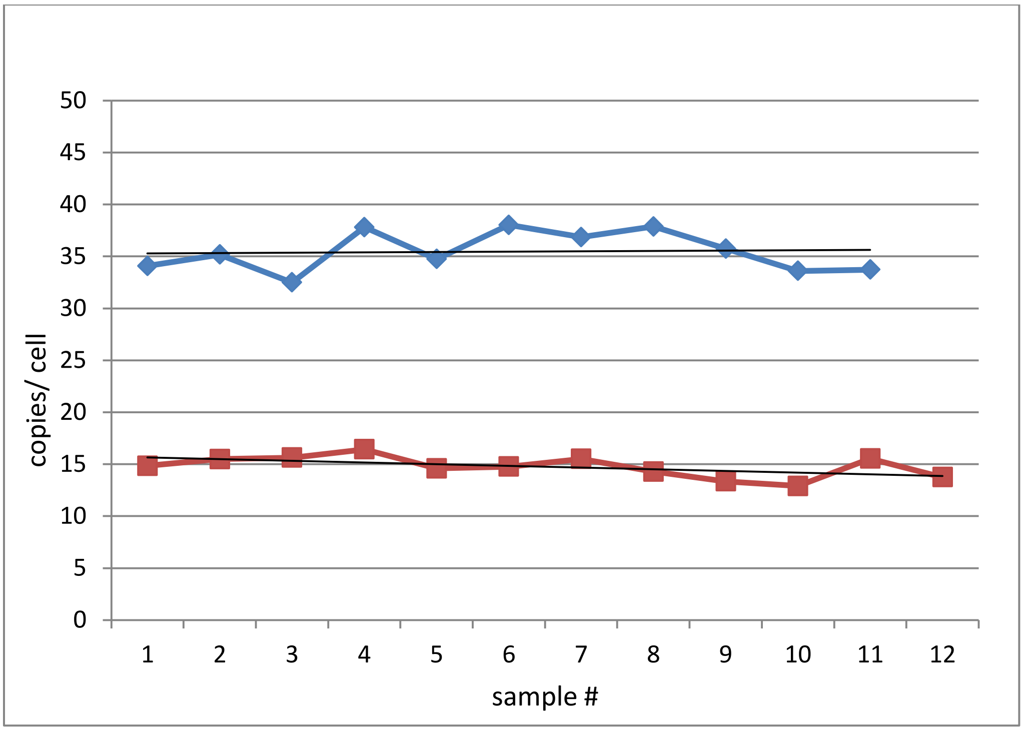

B

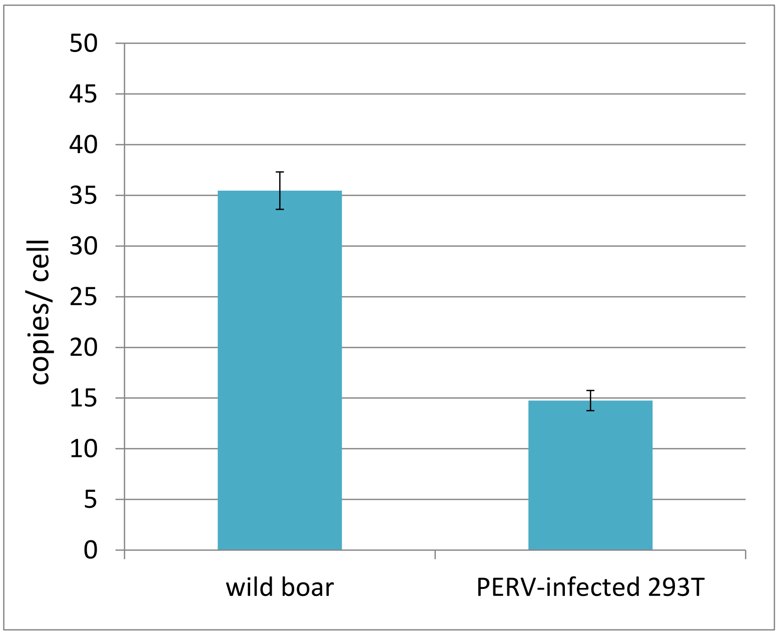

Supplemental Figure 2. Validation of the ddPCR. A, Eleven or 12 DNA samples, respectively, from the same DNA batch were used to determine the PERV copy number in wild boars (blue line) and PERV-infected 293 cells (red line) using ddPCR. B, Summary of the experiment shown in A, showing the mean value of the PERV copy number of the samples. In the wild boar samples 35.46 ± 1.85 copies/cell were detected, whereas the PERV-infected 293T samples showed 14.76 ± 1.0 copies/ cell.

Supplemental Table 1. Primers and probes used for the detection of PERV.

Primer, probe	Sequence	Accession number	Position (nt-nt)	Reference
PERV EnvA forward	5'-TGGAAAGATTGGCAACAGCG -3	Y12238	742-761	Tissier et al.,
PERV EnvA reverse	5'-AGTGATGTTAGGCTCAGTGG -3'		1101-1082	1997 [45]
PERV EnvB forward	5'-TTCTCCTTTGTCAATTCCGG -3'	Y12239	1376-1395	
PERV EnvB reverse	5'-TACTTTATCGGGTCCCACTG -3'		1639-1620	
PERV EnvC forward	5'-CCCCAACCCAAGGACCAG-3'		9601-9618	Kaulitz et al.,
PERV EnvC reverse	5'-AAGTTTTGCCCCCATTTTAGT-3'	AM229312	9692-9672	2011 [15]
PERV EnvC probe	5'-[6FAM] CTCTAACATAACTTCTGGATCAGACCC [BHQ1]-3'		9626-9652	
PERV pol forward	5'- CGACTGCCCCAAGGGTTCAA-3'	HM159246	3568-3587	Yang et al.,
PERV pol reverse	5'-TCTCTCCTGCAAATCTGGGCC-3'		3803-3783	2015 [8]
PERV pol probe	5'-[6FAM]CACGTACTGGAGGAGGGTCACCTG[BHQ1]-3'		3678-3655	
pactin forward	5'-TAACCGATCCTTTCAAGCATTT-3'			
pactin reverse	5'-TGGTTTCAAAGCTTGCATCATA-3'			
pactin probe	5'-[HEX]CGTGGGGATGCTTCCTGAGAAAG[BHQ1]-3'			
pGAPDH forward	5'- TTCACTCCGACCTTCACCAT -3'	396823	3951-3970	
pGAPDH reverse	5'- CCGCGATCTAATGTTCTCTTTC -3'		4022-4001	
pGAPDH probe	5'-[HEX]CAGCCGCGTCCCTGAGACAC[BHQ1]-3		3991-3972	

Reference 45: Le Tissier, P.; Stoye, J.P.; Takeuchi, Y.; Patience, C.; Weiss, R.A. Two sets of human-tropic pig retrovirus. Nature 1997, 389, 681–682.
